# Spleen stiffness: a predictive factor of dismal prognosis in liver cirrhosis

**DOI:** 10.1007/s12328-022-01752-z

**Published:** 2023-01-02

**Authors:** Dimitrios S. Karagiannakis, Katerina Stefanaki

**Affiliations:** 1 Academic Department of Gastroenterology, Medical School of National and Kapodistrian University of Athens, “Laiko” General Hospital, 17 Agiou Thoma Street, 11527 Athens, Greece; 2 Department of Clinical Therapeutics, School of Medicine, National and Kapodistrian University of Athens, “Alexandra” General Hospital, Athens, Greece

**Keywords:** Spleen stiffness, Liver decompensation, Portal hypertension, Esophageal varices, Survival

## Abstract

**Abstract:**

Portal hypertension (PH) is a major complication of liver cirrhosis, as it predisposes to the development of serious clinical manifestations such as ascites, hepatic encephalopathy and variceal bleeding, aggravating the prognosis of patients. Hepatic vein pressure gradient (HVPG) is considered the reference method for the estimation of the presence and severity of PH, but this procedure is available only in specialized centers. Alternatively, many non-invasive methods have been proposed in order to substitute HVPG. Among them, liver stiffness measurement (LSM) has been widely used, as it has been shown to correlate well with HVPG, though this relationship seems to weaken in values of HVPG higher than 12 mmHg, the threshold of serious complications development. Several studies supported the use of spleen stiffness measurement (SSM) instead of LSM, anticipating to a more adequate assessment of this advanced stage of PH. The aim of this paper is to critically appraise and summarize the literature about the role of SSM as a predictive tool of liver decompensation and prognosis, highlighting the strengths and the potential limitations of the studies published so far.

**Expert’s opinion:**

The utility of SSM in ruling out high risk for bleeding varices in cirrhotic patients has been demonstrated, driving the Baveno VII consensus to encompass SSM in its last recommendations, though its use in patients with non-viral cirrhosis remains to be validated. We believe that in the near future, SSM alone or combined with other tests, will being used not only for sparing upper endoscopies, but also for predicting decompensation and prognosis in advanced compensated cirrhotic patients, regardless of liver disease’s etiology. Herein, we present the data that support this consideration, pointing out these issues that should further be investigated in order to elucidate and intensify the value of SSM in the management of patients with liver cirrhosis.

## Introduction

Clinically significant portal hypertension (CSPH) constitutes a significant complication in patients with chronic advanced liver disease, leading to the formation of esophageal varices (EVs), ascites, hepatic encephalopathy (HE) and hepatorenal syndrome (HRS) [1, 2], comorbidities that contribute to increased morbidity and mortality [3]. Hepatic venous pressure gradient (HVPG) is the gold-standard method for the diagnosis of CSPH which is defined as values higher than 10 mmHg. Unfortunately, HVPG is an invasive and expensive procedure available only in specialized centers. Therefore, several non-invasive methods have been proposed in order to substitute HVPG for the evaluation of the severity of portal hypertension [3]. Several serum biomarkers and ultrasound Doppler parameters have been studied, but their diagnostic accuracy has been found to be suboptimal [4–7], while inter-observer and inter-equipment variability has also been noticed among different diagnostic centers [7–9]. Later on, liver stiffness measurement (LSM) using elastographic techniques was proposed as an adequate tool for the prediction of CSPH, as it was found to correlate well with portal pressure measured by HVPG [10, 11]. However, it has been documented that this correlation between LSM and HVPG weakens in HVPG values higher than 10–12 mmHg, which is the threshold for the development of severe liver complications. In addition, clear association between LSM and the size of EVs has not been verified [10–13]. These findings are not somewhat unexpected, as at the early stages of cirrhosis the main factor influencing the portal pressure is liver fibrosis, which is related to LSM [14], whereas at the later stages, the portal pressure mostly depends not on fibrosis, but on the increased portal vein inflow due to splanchnic vasodilation and the hyperdynamic circulation [15, 16]. It seems that changes in splenic density due to tissue’s hyperplasia, congestion, and fibrosis may reflect better this hyperdynamic component of portal hypertension [17]. The Baveno VI group adopted the combination of LSM by transient elastography (TE) with the platelets count, the latter as an indicator of hypersplenism, in order to discriminate patients with CSPH. According to that, advanced compensated cirrhotic patients with LSM < 20 kPa and platelets > 150,000/mm^3^ had a 95% possibility for not developing high-risk EVs, which is a common feature of CSPH. However, the percentage of cirrhotic patients fulfilling the above criteria avoiding an unnecessary upper endoscopy has been estimated close to 20–30%, making clear that in the majority of advanced cirrhotic patients, the detection of CSPH remains a disputable issue [18–23]. In order to increase the proportion of patients diagnosed with CSPH using non-invasive methods, some investigators proposed several other tests or algorithms. Among them, spleen stiffness measurement (SSM) alone or combined with other parameters such as LSM, seemed to provide encouraging results, attributed to the established correlation between the SSM and HVPG, clarified in many studies [24–26]. The aim of this review is to present the potential association between SSM and the risk of development of complications related to CSPH in patients with liver cirrhosis.

## SSM and formation of EVs and high-risk EVs

Many studies have shown the substantial ability of SSM to predict the existence of EVs. Stefanescu et al. reported that patients with SSM over 46.4 kPa had a higher probability to develop EVs (PPV 93.4%) [27]. Similarly, Fraquelli et al. found that SSM values up to 48 kPa were associated with absence of EVs in 100% of cases [28]. Furthermore, Sharma et al. demonstrated that SSM above 40.8 kPa, had sensitivity 94%, specificity 76%, PPV 91%, NPV 84% and diagnostic accuracy of 86% for predicting EVs [29]. All the above studies were based on TE for the evaluation of SSM. Notably, the cutoff values proposed in the first two studies were similar, while those of the third one slightly differed. This discrepancy can be explained by the fact that Sharma et al. included consecutive cirrhotic patients of any etiology, in contrast to the other studies where patients with viral hepatitis exclusively participated. A meta-analysis conducted in 2016 confirmed the excellent capability of SSM to detect the presence of EVs. Interestingly, the authors concluded that SSM is significantly superior to LSM in terms of identifying patients with EVs, with diagnostic accuracy 88% and sensitivity 88%, in comparison to accuracy 81% and sensitivity 83%, respectively [30]. Of note, the superiority of SSM over LSM was also ratified regarding the ability to predict the presence of high-risk for bleeding EVs [30] (Table 1).

**Table 1:** Spleen stiffness measurement for the prediction of EVs and high-risk EVs

References	Patients (*n*) etiology	Elastographic technique	Endpoint	Cutoff	Diagnostic performance
Stefanescu et al. [27]	135/all	TE	EVs	52.5 kPa	AUROC: 0.74
Sharma et al. [29]	200/all	TE	EVs	≥ 40.8 kPa	Se: 94%; Sp: 76%
			High-risk versus low-risk EVs		PPV: 91%; NPV: 84%
			56 versus 49 kPa (*p* = 0.001)		86% accuracy
			Bleeding versus no bleeding		
			58 versus 50.2 kPa		
			(*p* = 0.001)		
Takuma et al. [34]	340/all	pSWE	EVs	3.18 m/s	NPV: 98.4%; Se: 98.5%, 75% accuracy
			High-risk EVs	3.30 m/s	NPV: 99.4%; Se: 98.9%, 72.1% accuracy
Fraquelli et al. [28]	132/viral	TE	EVs	48.5 kPa	NPV: 100%
Kim et al. [40]	106/all	pSWE	Hemodynamic response to NSBBs by ΔSSM model	0.53	Se: 81.4%; Sp: 74.5%; positive-LH: 3.192; negative-LH: 0.25; accuracy: 0.783
					AUROC: 0.801
Fierbinteanu-Braticevici et al. [33]	135/all	pSWE	EVs versus no EVs		
			3.37 m/s versus 2.79 m/s		
			(*p* < 0.001)		
			High-risk versus low-risk EVs		
			3.96 m/s versus 2.93 m/s		
			(*p* < 0.001)		
Karagiannakis et al. [35]	71	2D-SWE	High-risk EVs	< 33.7 kPa	NPV: 92.3%
					AUROC: 0.792
					Misclassification: 3.1%
Hu et al. [36] (meta-analysis)	3952	All	EVs		Pooled Se: 90%, Sp: 73%
					AUROC: 0.87
	32 studies		High-risk EVs		Pooled Se: 87%, Sp: 66%
					NPV: 88%, PPV: 54%
					AUROC: 0.83

TE, Transient elastography; pSWE, point shear wave elastography; 2D-SWE, 2-dimension shear wave elastography; EVs, esophageal varices; NSSBs, non-selective b-blockers; PPV, positive predictive value; NPV, negative predictive value; Se, sensitivity; Sp, specificity; LH, likelihood; AUROC, area under receiver-operating curve

Currently, the good diagnostic performance of SSM on identifying patients with EVs was demonstrated, even when other elastographic techniques apart from TE were applied [31, 32]. A recent study, which evaluated SSM using point shear wave elastography (pSWE), underlined that regardless of liver disease’ etiology, patients with EVs of any grade had significantly higher average SSM values compared to those with no EVs (3.37 m/s vs. 2.79 m/s, *p* < 0.001), while in cases of high-risk EVs, the difference was even greater (3.96 m/s vs. 2.93 m/s, *p* < 0.001) [33]. These findings are in agreement with the results of a previous study based on the same elastographic method, which proved the superiority of SSM over any other non-invasive parameter and clarified that a cutoff value < 3.18 m/s might securely exclude the existence of EVs (NPV 98.5%), whereas values < 3.30 m/s could rule out the presence of high-risk EVs (NPV 99.4%) [34].

Our group recently performed two-dimension shear wave elastography (2D-SWE) in cirrhotic patients and assessed the capability of SSM in ruling out high-risk EVs. Once more, the diagnostic accuracy of SSM was confirmed, as in the total cohort of patients, SSM values < 33.7 kPa were found to almost exclude the presence of high-risk EVs with AUROC 0.792 and NPV 92.3%. The misclassification rate was 3.1%, and 40.6% of patients could skip an upper endoscopy [35]. In a recent meta-analysis of 32 studies and 3952 patients, the pooled sensitivity and specificity of SSM in detecting EVs were 90% and 73%, respectively, with an AUROC of 0.87. Furthermore, the pooled sensitivity, specificity, PPV and NPV of SSM in detecting high-risk EVs were 87%, 66%, 54% and 88%, respectively, with an AUROC of 0.83. Considering the pooled NPV and the prevalence of high-risk EVs in the included studies, 50.6% patients could avoid endoscopy, with a risk of missing high-risk EVs of 8.4%. Notably, in this meta-analysis, the selected studies had been based on different elastographic techniques [36]. Thiele et al. had previously published a meta-analysis of 5 studies which had been performed by solely using 2D-SWE. Data obtained from 328 individual patients and the SSM cutoff value of 14 kPa offered a high sensitivity (91%), but also a modest specificity (37%), in ruling out CSPH. Albeit, a high false-negative rate was reported, as 60% of patients with SSM below 14 kPa had CSPH. The high false-negative rate was attributed to the high proportion of decompensated patients who had been enrolled. In contrast, only 3% of patients with SSM above the rule-in CSPH cutoff 32 kPa were false positive. A substantial proportion of patients, almost the one-third, had SSM between 14 and 31.9 kPa and, therefore, could not be classified. Furthermore, the authors reported that 74% of patients in the gray zone had varices needing treatment (74%). The discrepancy between the results of this meta-analysis and those formerly provided underlines the difficulty of designing decision-making tools when not only patients with advanced compensated but also with decompensated disease are pooled [37] (Table 2).

**Table 2:** SSM as a predictor of liver decompensation and survival in cirrhotic patients, as well as in cases of TIPS implementation or resection for HCC in particular

References	Patients and etiology	Elastographic technique	Endpoint	Cutoff	Diagnostic performance
Jansen et al. [43]	158/all	2D-SWE	Rule out CSPH	LSM: 16 kPa	Se: 98.6%; Sp: 70.3%
				+	PPV: 86.6%; NPV: 96.3%
				SSM: 26.6 kPa	89% accuracy
Colecchia et al. [45]	124/HCV	TE	Liver decompensation at 2 years	54 kPa	NPV: 97.5%
Takuma et al. [46]	393/all	pSWE	Liver decompensation	3.25 m/s	NPV: 98.8%
			Death	3.43 m/s	68.9% accuracy
					NPV: 9.3%
					75.8% accuracy
Wu et al. [50]	54/all	TE	PLF after hepatectomy for HCC		No significant difference in SSM between patients with and without PLF (*p* = 0.36). No significant difference in OS was observed between patients with an SSM of < 22.3 and ≥ 22.3 kPa (*p* = 0.378)
Meister et al. [47]	210/all	TE	Liver decompensation at 1 year	39 kPa	Se: 100%; Sp: 69%; NPV: 100%; PPV: 21%
					AUROC: 0.91
Marasco et al. [51]	175/all	TE	Late recurrence (> 24 months) of HCC after resection	70 kPa	NPV: 75%; PPV: 75%
			Late recurrence-free survival	70 kPa	Higher in SSM < 70 kPa vs > 70 kPa (*p* = 0.0002)
Zhu et al. [49]	89/all	pSWE	Death after TIPS	3.6 m/s	Se: 54.2%; Sp: 90.8%
					NPV: 84.3%; PPV: 68.5%
					80.9% accuracy
Karagiannakis et al. [48]	177/all	2D-SWE	Liver decompensation at 1 year	37 kPa	NPV: 81.1%
					AUROC: 0.71
			Death/liver transplantation at 1 year	38.8 kPa	NPV: 95%
					AUROC: 0.72

SSM, Spleen stiffness measurement; TE, transient elastography; pSWE, point shear wave elastography; 2D-SWE, 2-dimension shear wave elastography; PLF, post-liver failure; TIPS, transjugular intrahepatic portosystemic shunt; HCC, hepatocellular carcinoma; PPV, positive predictive value; NPV, negative predictive value; Se, sensitivity; Sp, specificity; AUROC, area under receiver-operating curve

In order to increase the prediction ability for EVs and high-risk EVs, reducing the need for an upper endoscopy, some investigators tried to combine LSM with SSM. Wong GH et al. first demonstrated in a randomized study, the possible non-inferiority of a screening strategy for EVs, guided by LSM/SSM combination, compared to universal endoscopic screening in detecting CSPH. Subsequently, they prospectively followed up the recruited patients, with primary end-point the incidental variceal bleeding confirmed with an upper endoscopy. It was a non-inferiority, open-label, randomized, controlled trial of 548 adult patients with compensated liver cirrhosis. During the observation period, 4.4% in the LSM/SSM arm and 4% in the conventional arm developed variceal bleeding (log-rank test *p* = 0.724). The incident rates of hepatic events were also similar in both arms (*p* = 0.327). Consequently, the authors supported that the initiation of the LSM/SSM strategy in clinical practice could safely save up to half of the upper endoscopies [38, 39].

Except from the established SSM’s role in ruling out patients with EVs and high-risk EVs, SSM may also estimate the response to treatment with non-selective b-blockers (NSBB). Kim and al. showed that when a hemodynamic response to NSBB occurred, ΔSSM (reduction in SSM) was the only parameter independently associated with that response. The authors displayed a prediction model based on ΔSSM and reported a good predictive performance, with AUROC 0.803 for the threshold value of 0.530. These findings were further confirmed by the same researchers in a validation set [40].

## SSM and development of liver decompensation events

Until now, SSM has not been adequately investigated regarding its potential utility in predicting future decompensation events in patients with advanced compensated cirrhosis. Remarkably, most of the studies have assessed the accuracy of LSM compared to HVPG. Indeed, Jindal et al. elucidated a time-dependent AUROC (over 5 years of follow-up) of 0.716–0.742 and 0.709–0.784, for predicting liver decompensation by measuring HVPG or liver stiffness, respectively. A LSM value up to 22 kPa was found to offer a 90% NPV for incident all-cause decompensation event [41]. In order to identify a larger number of patients being at potential risk for liver decompensation, Trebicka et al. combined LSM with MELD score. Among 1827 patients evaluated by 2D-SWE, they showed that a LSM cutoff at ≥ 20 kPa combined with MELD ≥ 10 could stratify the risk of first decompensation event, with a 2-year decompensation rate of 61.8% in the high-risk group (LSM ≥ 20 kPa plus MELD score ≥ 10) compared to 3.5% in the low-risk group [42].

Jansen et al. verified that the addition of SSM to LSM could further contribute to the detection of patients eligible for developing decompensation. The authors introduced a rule-out and a rule-in algorithm. According to them, LSM < 16 kPa was able to rule out CSPH, with a sensitivity of > 90%. Cutoff values of LSM > 29.5 kPa were able to rule in CSPH with a specificity of > 90%. On the other hand, SSM < 21.7 kPa was able to rule out CSPH with a sensitivity of 91.9%, while values of SSM > 35.6 kPa were able to rule in CSPH, with a specificity of > 90%. The combination of LSM > 29.5 kPa and SSM > 35.6 kPa was able to rule in CSPH, with specificity > 92% [43, 44].

Colecchia et al. had first shown solely in HCV cirrhotic patients, that during a 2-year follow-up period, the only factors independently associated with the occurrence of clinical decompensation events were the SSM by TE and the MELD score. The authors initiated a predictive model including SSM and MELD score with an excellent discriminative ability, as the C-index was 0.87, while for the model including SSM alone was 0.85. The C-index for the model including only HVPG was 0.83, which was not statistically different from those of SSM plus MELD and SSM alone models. Considering the simplified model including only SSM, patients with values lower than 54 kPa were at low risk of events at 2 years, with a 97.5% NPV [45]. Later on, Takuma et al. estimated the accuracy of SSM using pSWE, on predicting liver decompensation events in cirrhotic patients, regardless of the etiology of liver disease. The authors identified SSM and MELD score as the only factors independently associated with decompensation [SSM: HR, 14.500 (95% CI 4.970–42.300), *p* < 0.001; MELD: HR, 1.196 (95% CI 1.063–1.345), *p* = 0.003]. Furthermore, SSM offered the highest C-index for predicting hepatic decompensation, with statistically significant differences between the C-index of SSM and those of MELD (*p* = 0.006), Child–Pugh score (*p* = 0.002) and LSM (*p* < 0.001). The SSM cutoff value of 3.25 m/s had a NPV of 98.8% and accuracy 68.9% in predicting hepatic decompensation [46].

Recently, Meister et al. investigated patients with liver disease of any etiology and reported that those who developed decompensation had SSM values by TE higher than 39 kPa [47]. In the study of Karagiannakis et al., similar results were obtained, as regardless of liver disease’s etiology, SSM was found as the only factor independently associated with the probability of decompensation (HR: 1.063, 95% CI 1.009–1.120; *p* = 0.021). Moreover, the predictability of SSM was superior compared to LSM and MELD score, offering an AUROC of 0.710 (*p* = 0.003), while patients with SSM < 37 kPa had < 20% probability for developing decompensation during the next 1 year of follow-up (sensitivity 74.1%, specificity 72.7%, NPV 81.1%). It has to be mentioned that the above studies had not used the same elastographic methods for the assessment of SSM [48].

## SSM and survival

To date, only a small number of studies have reported an association between SSM and survival in cirrhotic patients. Takuma et al. in the study previously referred, apart from the efficacy of SSM to predict decompensation, they also investigated the association between SSM and patients’ survival. According to their results, SSM showed the highest C-index in predicting mortality compared to LSM and MELD score, not only to compensated, but also to decompensated patients. A SSM cutoff value of 3.43 m/s had a NPV of 95.3% and accuracy of 75.8% for predicting mortality in all patients. In addition, the optimal SSM cutoff value separately in compensated and decompensated patients was 3.41 m/s and 3.53 m/s, respectively. Importantly, SSM was an independent parameter associated with mortality after adjustment for ALT, serum sodium, and MELD score in the Cox regression analysis and each SSM unit (m/s) increase was associated with a 14.5-fold increase in the risk of death [46]. Karagiannakis et al. recently confirmed the superiority of SSM over LSM, MELD score and the combination of both LSM and MELD score, in predicting transplantation-free survival in cirrhotic patients. In the Cox regression analysis, only SSM was found to be independently associated with death or liver transplantation. Although SSM offered a moderate predictability of 1-year death or liver transplantation (AUROC: 0.72), patients with SSM < 38.8 kPa had a 95% probability of transplantation-free survival during the next 1 year. Another important finding of this study was the maintenance of SSM predictivity even among patients potentially being at the highest risk of a poor outcome, i.e., those with Child–Pugh class B or C plus MELD > 10 plus LSM > 20 kPa. In this subgroup, the predictability of SSM was even more potent, as it increased from moderate to good (AUROC: 0.80), whereas 96% of patients with SSM < 38.8 kPa survived without liver transplantation during the 1 year of follow-up [48].

The association between SSM and survival has also been appraised after the insertion of TIPS, or postoperatively after hepatic resection for hepatocellular carcinoma (HCC). In a retrospective cohort study, which used pSWE as screening method for consecutive patients who underwent transjugular intrahepatic portosystemic shunt (TIPS), the independent prognostic factors of survival after TIPS implementation were the SSM, the LSM, the diameter of shunt and the older age. SSM particularly correlated with liver failure after TIPS insertion, with a 57.440-fold rise in the risk of death for each SSM unit (m/s) increase. A SSM value of 3.60 m/s could predict survival after TIPS, with a sensitivity of 54.2% and specificity of 90.8% [49].

The efficacy of preoperative LSM and SSM measured by TE, in predicting the risk of postoperative liver failure (PLF) after hepatic resection for HCC, was evaluated by Wu et al. The authors observed that patients with PLF had higher preoperative LSM compared to those without, while no significant difference was noticed regarding the SSM [50]. This could probably be explained by the fact that LSM seems to express better the liver capacity and competency that affect the prognosis at the early post-hepatectomy stage, while SSM which expresses better the portal hypertension, probably affects the mortality in the later post-surgical periods. Furthermore, the small number of patients developed PLF (7/54, 13%), which leads to results with limited prognostic value. Currently, in patients with HCC surgically resected, SSM seemed to be a predictor of late (> 24 months after the procedure) HCC recurrence. In an Italian study, early HCC recurrence at multivariate analysis was associated with viral etiology, HCC grading (3 or 4), resection margins < 1 cm and being beyond the Milan criteria. On the other hand, late HCC recurrence at univariate analysis was associated with EVs, spleen length, platelet/spleen length ratio, LSM and SSM. In the multivariate analysis, only SSM was independently associated with late recurrence (HR 1.046, CI 1.020–1.073). Obviously, the early relapse of HCC depends on factors related to the biological characteristics of the tumor, while the late recurrence appears to be related with parameters that mainly represent the severity of liver failure and portal hypertension [51].

## Where are we now? Concerns, limitations, future perspectives, experts’ opinion

The advantage of SSM in comparison to LSM is its ability to reflect better the more advanced stages of PH, where the splanchnic vasodilation predominates. In addition, the accuracy of SSM is not affected by factors that influence the diagnostic performance of LSM, such as the presence of liver inflammation, necrosis and probably steatosis, or the existence of comorbidities, such as cholestasis, vascular congestion and right cardiac failure [52]. Certainly, the practice guidelines that have been proposed for optimal performing of LSM must be similarly followed and applied in order to achieve high accuracy and reproducibility of SSM as well [52].

The role of SSM for the evaluation of cirrhotic patients with CSPH has been clearly shown, specifically its accuracy to detect or rule out patients with EVs and high-risk EVs. Nonetheless, as studies’ methodology varied (i.e., different study population, use of different elastographic methods), a common validated SSM cutoff value for ruling in or ruling out EVs and high-risk EVs is still unclear. Taking on account the data published so far, the last BAVENO VII meeting recommended that: SSM by TE can be used in patients with advanced compensated liver disease, only due to viral hepatitis (untreated HCV; untreated and treated HBV), to rule out and rule in CSPH (SSM < 21 kPa and SSM > 50 kPa, respectively). In patients who are not eligible for NSBBs treatment (contraindication/intolerance) and are candidates for an upper endoscopy according to the Baveno VI criteria, (LSM by TE ≥ 20 kPa and/or platelet count ≤ 150 × 10^9^L), SSM ≤ 40 kPa by TE can differentiate those at low probability for having high-risk EVs, in order to avoid an endoscopy. Further validation of SSM in cirrhosis of other etiology except from viral hepatitis is required [53]. In addition, validation of the best cutoff values for pSWE and 2D-SWE is needed, as well as for the new 100 Hz specific spleen-dedicated TE module. The latter was recently introduced, in order to increase the feasibility of TE and seems to be a reliable, feasible and high reproducible tool, providing low rates of inter-observer variabilities [54, 55]. According to a recent study, the success rate of SSM was higher with the 100 Hz module compared to the original of 50 Hz (92.5% vs. 76%, respectively, *p* < 0.001). Moreover, the former had higher accuracy in diagnosing high-risk EV’s (AUROC 0.782 vs. 0.72, respectively, *p* = 0.027), while this was higher even when compared to those of LSM (AUROC of LSM: 0.615) [56]. Notably, in order to increase the feasibility of SSM by TE, other investigators tried to use the controlled attenuation parameter (CAP) as a guiding tool. Interestingly, CAP showed a good predictivity of appropriate SSM performance. Particularly, in cases of CAP values lower than 118 db/m, the correlation of SSM with HVPG was significantly better, as well as the accuracy of SSM in predicting high-risk EVs [57].

Regarding SSM as a predictor of liver decompensation events and survival, more and larger studies are necessary. Though the number of participants in current studies is not negligible, the proportion of those altering from a compensated to a decompensated state is certainly not sufficient enough for making definite extrapolations. Furthermore, it has to be clarified which are the optimal cutoff values for predicting decompensation and survival, concerning the liver disease’s etiology and the elastographic modality being used. In addition, it shall be investigated more thoroughly whether the initiation of treatment (i.e., NSBB administration) changes the results of SSM and how this reflects on the decompensation risk and prognosis of patients.

In conclusion, to summarize, SSM seems to correlate better with CSPH compared to LSM. Lot of studies have emerged this superiority, leading to the recent modified Baveno criteria that include the SSM in the predictive algorithm of cirrhotic patients at low probability for high-risk EVs. Nevertheless, as liver function deteriorates and portal hypertension aggravates, the probability of development of a decompensated event increases and prognosis worsens. Although there are some promising results about the effectiveness of SSM in predicting decompensation and outcome in cirrhosis, further validation is required. Additional studies, with larger sample of patients of similar characteristics (i.e., same etiology of liver disease), evaluated by the same elastographic technique must be carried out in order to ascertain whether SSM, alone or in combination with other tests, might play a significant and undoubted role in differentiating patients at risk for complications and which could be the most ratified SSM cutoff value for this challenge.

## Abbreviations

CSPH: Clinically significant portal hypertension
EVs: Esophageal varices
HE: Hepatic encephalopathy
HRS: Hepatorenal syndrome
HVPG: Hepatic vein pressure gradient
LSM: Liver stiffness measurement
TE: Transient elastography
SSM: Spleen stiffness measurement
NPV: Negative predictive value
PPV: Positive predictive value
pSWE: Point shear wave elastography
2D-SWE: Two-dimension shear wave elastography
AUROC: Area under roc curve
NSBB: Non-selective b-blockers
MELD: Model for end-stage liver disease
HR: Hazard ratio
TIPS: Transjugular intrahepatic portosystemic shunt
HCC: Hepatocellular carcinoma
PLF: Postoperative liver failure
